# Characterization of SARS-CoV-2 recombinants and emerging Omicron sublineages

**DOI:** 10.7150/ijms.79116

**Published:** 2023-01-01

**Authors:** Yuliang Wang, Yiyin Long, Feng Wang, Changlin Li, Wei Liu

**Affiliations:** 1 Tianjin Institute of Urology, The Second Hospital of Tianjin Medical University, Tianjin, China; 2 Department of Genetics, School of Basic Medical Sciences, Tianjin Medical University, Tianjin, China; 3 Tianjin Children's Hospital, Children's Hospital, Tianjin University, Tianjin, China

**Keywords:** COVID-19, SARS-CoV-2, Omicron, recombinant, BA.5, vaccine

## Abstract

The SARS-CoV-2 Omicron is currently the predominant circulating variant in the COVID-19 pandemic. The dominating Omicron sublineages respond to host immune pressure and develop advantageous mutations or genetic recombination, which result in variants that are more contagious or better at escaping immune responses in response to previous infection or vaccination. Meanwhile, multiple genetic recombination events have been reported in coinfection cases, the majority of which have resulted from the recombination between co-circulating Omicron BA.1 (or BA.1.1) and Delta variant or BA.2. Here, we review the knowledge and characterization of recombination for SARS-CoV-2 at the population level, provide an update on the occurrence of newly circulating Omicron sublineages, and discuss the effectiveness of novel vaccines/therapeutic drugs against the Omicron variant.

## Introduction

The ongoing COVID-19 pandemic is fuelled by the highly infectious severe acute respiratory syndrome coronavirus 2 (SARS-CoV-2) Omicron variant, which was first reported in Botswana and quickly spread as the dominant variant worldwide during early 2022 1. Throughout COVID-19 pandemic, SARS-CoV-2 cases and subsequent hospitalizations, driven by different variants at various time points and currently Omicron sublineages, have been on a rise. So far, the mutating Omicron has evolved into distinct sublineages and descendent lineages that are characterized by more than 30 mutations in the spike (S) protein compared to the ancestral wild type strain (WT). This has not only strengthened the infectivity/transmissibility of these variants, but also compromised the protection provided by vaccines or humoral immunity induced by prior natural infections 2,3. In hosts with immunocompromised status, such as those who have received organ transplantation, have HIV or cancer, or are undergoing medical treatments like chemotherapy, SARS-CoV-2 infection lasts significantly longer than previously recognized. For example, a case report on an asymptomatic immunocompromised individual with cancer described prolonged infection with SARS-CoV-2 shedding for up to 105 days 4. Another report on an immunocompromised individual, who was infected with ten distinct SARS-CoV-2 variants including several major variants of concern (VOCs), Alpha, Gamma, and Omicron, described prolonged infection with SARS-CoV-2 and virus shedding for up to 505 days before passing away 5. The average rate of coinfection between dominant VOCs, although low, tended to increase with the increased diversity of SARS-CoV-2 during continuing epidemic waves 6. Moreover, considering the long-lasting waves of SARS-CoV-2 infections globally, patients with prolonged coinfection form a critical cohort that has a higher possibility of facilitating accumulations of S mutations and genetic recombination over time, which would result in novel SARS-CoV-2 recombinants of unknow properties 7,8. Here, we review the knowledge regarding characterization of Recombination in SARS-CoV-2 at a population level, provide an update on the occurrence of newly circulating Omicron sublineages, and discuss effectiveness of novel vaccines/therapeutic drugs against the Omicron variant.

## Recombination in SARS-CoV-2

With the progression of COVID-19 pandemic and evolution of genetically divergent SARS-CoV-2 lineages/sublineages, viral recombinants harbouring mutations acquired from distinct lineages or sublineages to reshape SARS-CoV-2 genetic diversity that confer pathogenic properties distinct from the parental lineage, are becoming a major challenge 8-18. Vaninsberghe et al. 13 identified 1,175 putative recombinants within the first year of SARS-CoV-2 circulation after analysing the 537,360 complete SARS-CoV-2 genome sequences available on Global Initiative on Sharing All Influenza Data (GISAID). They also estimated that not more than 0.2-2.5% of circulating SARS-CoV-2 strains in the USA and UK were recombinant. During this period, another study added further 221 candidates' recombinant lineages to the five proposed in the study by Vaninsberghe et al. and sequenced 225 likely recombinant genomes out of 87,695 complete genomes of SARS-CoV-2 8. Similarly, Turkahia et al. proposed 589 recombinant events (43,104 descendant samples) following collection of 1.6 million SARS-CoV-2 sequences, which indicated approximately 2.7% of the sequenced SARS-CoV-2 genomes belonged to the detectable recombinant lineages 15.

In this scenario, nomenclature of SARS-CoV-2 variants has become more important than ever. GISAID, Nextstrain, and Pango are the three most common nomenclature systems used during the COVID-19 pandemic. Additionally, WHO established an easier and more practical naming scheme using Greek letters to name the variants of interest (VOIs) and variants of concern (VOCs) for public communication 19. The Pango nomenclature system begins with a prefix “X” to designate all top-level recombinant lineages and follows usual suffixing rules to name their non-recombinant descendent lineages 20. Moreover, the system designates a new recombinant lineage only when it harbours a complete or near-complete SARS-CoV-2 genome, which may lead to fewer recombinant lineages designated as Pango lineages than their actual number 21.

**Figure 1:**
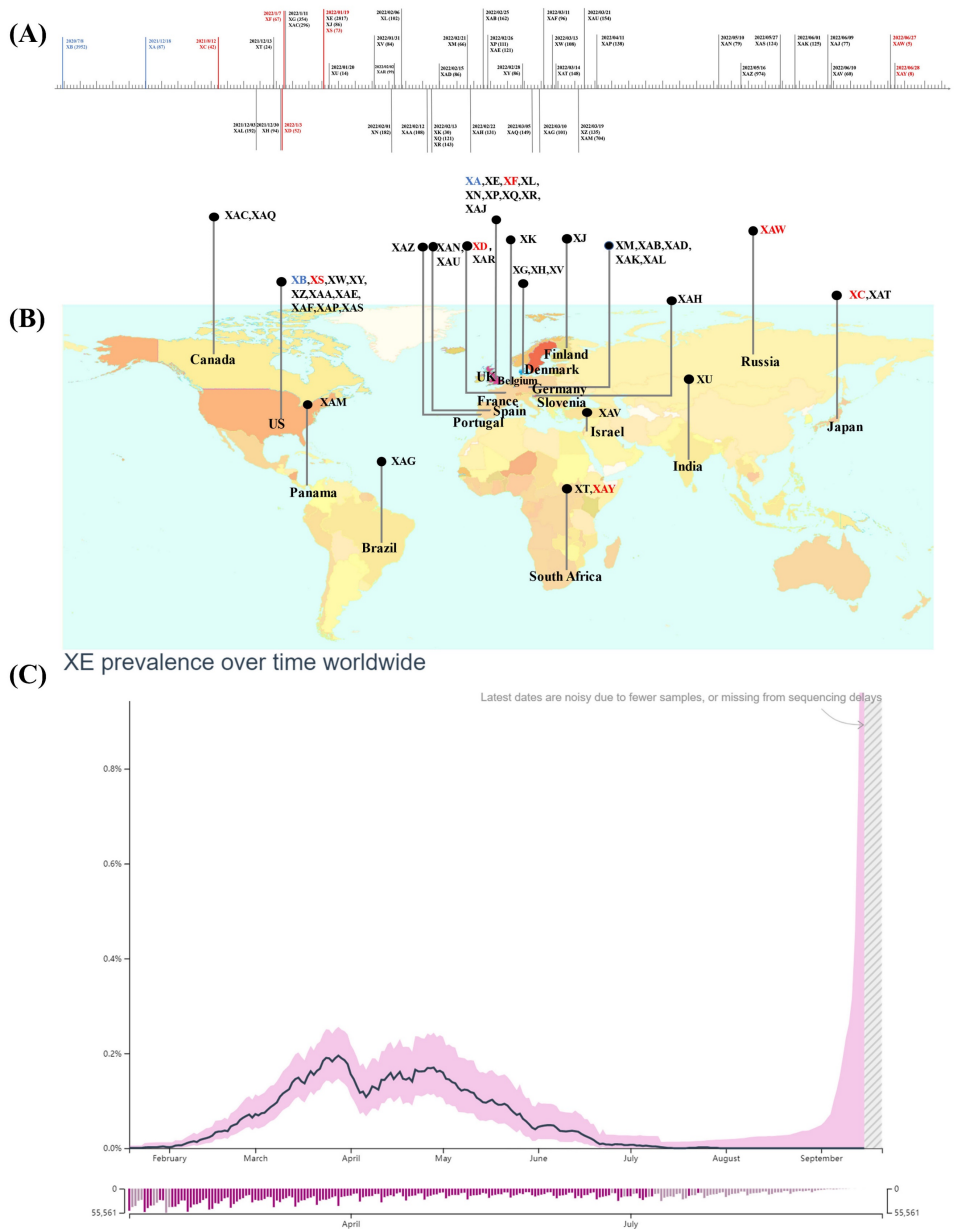
Emergence and spread of recombinant Pango lineages. Data obtained and modified from associated references and GISAID (accessed on 20 September 2022). **(A)** Sample collection date timeline and the numbers of cases designated and assigned for recombinant Pango lineages. **(B)** The top circulating country for each recombinant Pango lineage. The inter-VOC recombinant lineages are highlighted in red, intra-Omicron recombinant lineages are represented in gray, and others are in blue. **(C)** Average daily XE prevalence globally.

Notably, lineage XA, the first true Pango lineage recognized in the UK on 18^th^ December 2020, was only assigned to the Pango nomenclature system in May 2021 following collection of recombinant sequences between the parental lineages of B.1.1.7 and B.1.177 22. Besides, the circulating recombinants of B.1.631 and B.1.634 were designated as lineage XB, by the Pango nomenclature system in 2021, they showed a larger and more extensive circulation compared to XA 23. Subsequently, another 44 recombinant lineages were designated using the Pango nomenclature system **(Figure 1A, B)** 24, and the majority of these arose after the appearance of the Omicron variant, resulting in recombination between co-circulating Omicron sublineages BA.1(or BA.1.1) and Delta variant or BA.2 25.

### Inter-VOC recombinations

**Figure 2:**
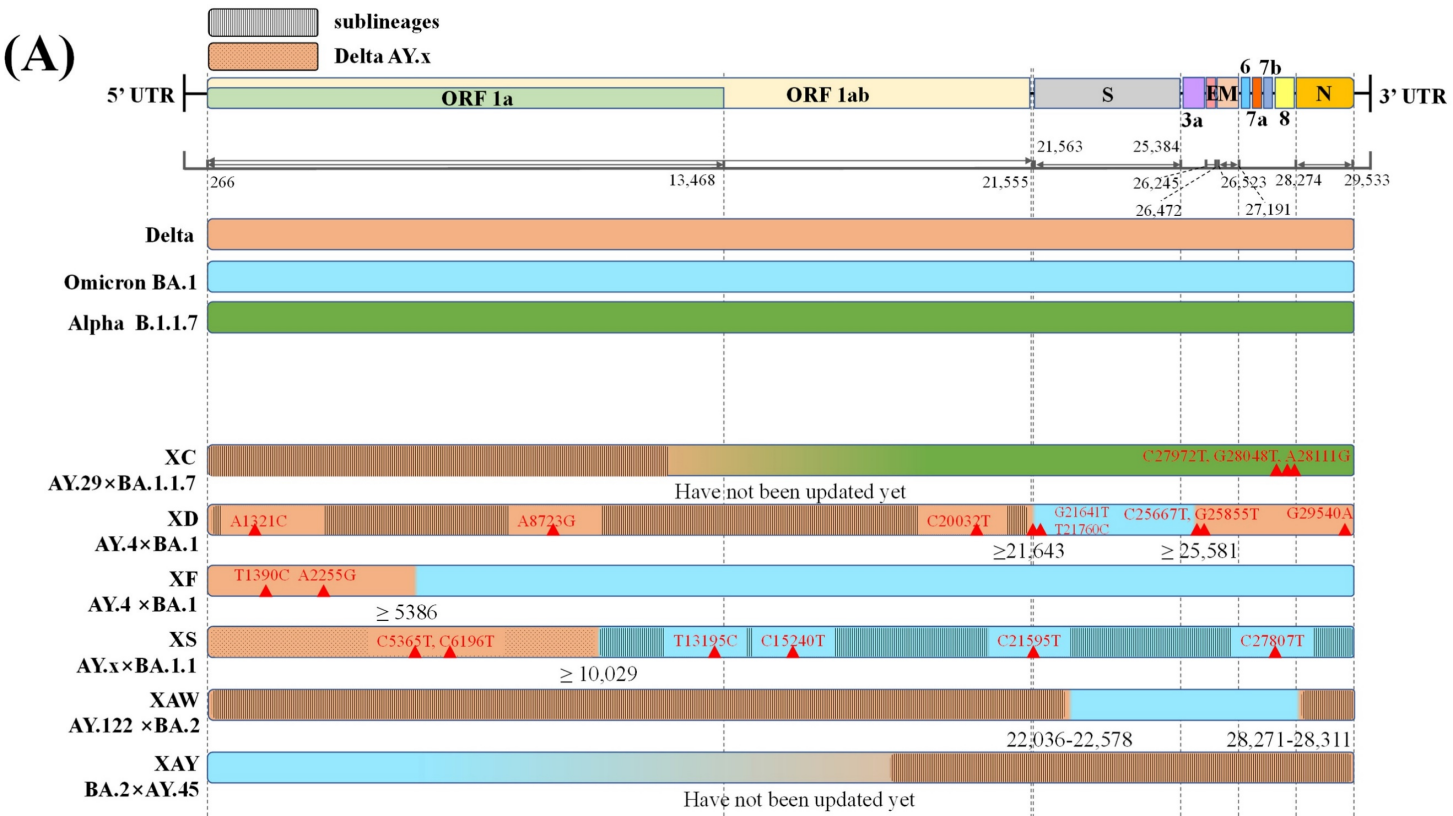

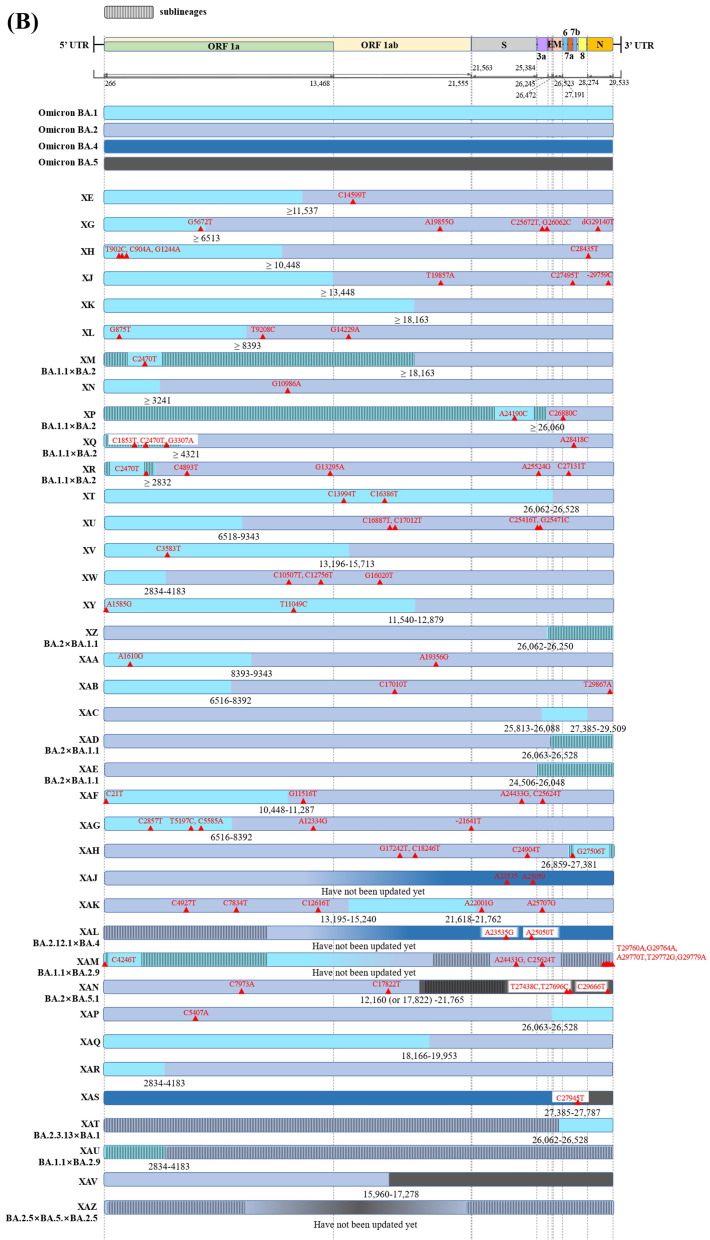
Graphical illustration of genenoms comparison in **(A)** inter-VOC and **(B)** intra-Omicron recombinants. Data obtained and modified from GISAID and associated references. In Figure 2 (A) or (B), the first part shows SARS-CoV-2 linear genome architecture and locations of encoded viral protein. The second part shows different parent lineages of recombinant Pango lineages with different colors. Different recombinant Pango lineages are in part 3 colored differently, annotated with break points and the potential sites or ranges of their break points. Unlabeled private mutations and locations of recombinant Pango lineages are depicted using red triangles and figures.

Only six recombinant Pango lineages are between SARS-CoV-2 VOCs, comprising one recombinant lineage between Delta AY.29 and Alpha and five recombinant lineages between Delta and Omicron variants **(Figure 2A)** 24,26. The mosaic genome structures shown in **Figure 2A** suggest that Delta variants tend to retain the 5'-terminal of the SARS-CoV-2 genome while Omicron or Alpha preserves the 3'-terminal during recombination. The only recombinant Pango lineage XC between Delta AY.29 and Alpha was reported by Sekizuka et al., but it showed limited circulation, with evidence of only a small cluster circulation in Japan 27,28.

In contrast, multiple putative Delta-Omicron recombinants have been reported so far 29-31. Previously, these recombinants featured elements of both parental lineages, the highly pathogenic Delta variant and, the highly transmissible Omicron variant, thus they were nicknamed "Deltacron" by Kostrikis et al. 32. Presently, several scientists use this name 33, while others refer to “Deltacron” only as recombinant lineages between Delta AY.4 and Omicron BA.1 34. Notably, the first Delta-Omicron recombinant virus was confirmed after the genome sequence was submitted to GISAID by Institut Pasteur in France on the 17^th^ January 2022 (EPI_ISL_10819657 incl. raw reads) 33,35. Subsequently, it was classified as clade GKA by GISAID, designated as XD by the Pango nomenclature system, and listed as a variant under monitoring (VUM) on the 9^th^ March 2022 by WHO 36,37. Genetically, XD has two likely breakpoints, one in 21,643 bp in S protein encoding gene and another in 25,581 bp at the beginning of the open reading frame (ORF) 3a 38. XD contains a large portion of the S gene from Omicron BA.1 and other genes from Delta AY.4 as its genomic backbone 33. Consistently, Simon-Loriere et al. 39 reported that XD behaviour was more similar to Delta in mice expressing human ACE2 receptors, but its immune escape properties were similar to those of Omicron BA.1. In addition, XD, presented a replicative advantage in the upper airways of K18-hACE2, but was outcompeted in the lungs when compared with Omicron BA.1 39. Furthermore, it was characterised by eight unlabelled private mutations **(Figure 2A)**. XD variant was also reported in countries like Denmark and the Netherlands 37.

By contrast, another recombinant lineage XF between Delta AY.4 and Omicron BA.1 harbours a higher portion of genome from the Omicron BA.1 variant. XF; it was first detected on 7^th^ January 2022 in the UK and was monitored by the UK Health Security Agency (UKHSA). Its non-structural protein (NSP) 1-3 is encoded by the genome acquired from Delta AY.4, while S protein and structural protein encoding genes are acquired from BA.1; moreover, it harbours a break point near the end of NSP3 (nucleotide 5,386). Regarding infectivity, the XF showed no specific age and sex predilections and infected a small cluster of 39 cases in the UK during 7^th^ January 2022 to 14^th^ February 2022, and has emerged and transmitted in at least eight other countries 38,40.

### Intra-Omicron recombinations

38 recombinant Pango lineages are evident in **Figure 2B**, of these 32 are recombinants of BA.1 (or BA.1.1) and BA.2 with six being recombinants of other Omicron sublineages. In all but eight of the 32 recombinant Pango lineages, BA.1 (or BA.1.1) retains the 5'-terminal of the SARS-CoV-2 genome while BA.2 preserves the 3'-terminal **(Figure 2B)**. Of the recombinant lineages, XJ harbors a breakpoint at the end of ORF1a without any novel mutations. Besides, a study reported at least 191 confirmed cases of XJ in Finland between week three and 11 of 2022, thus displaying community transmission and virus dispersion beyond borders 41. Another mutant hybrid XN harbours a likely recombination site located at nucleotide 3,241 on the NSP3; the recombinant lineage was originally reported in the UK in the first of February 2022 and then transmitted to several countries including Denmark, the USA, and Italy 42,26.

Notably, although only a few hundred sequences of other recombinant lineages of BA.1 (or BA.1.1) and BA.2 are available in GISAID so far, more than 2,500 sequences were reported for XE. XE was sequenced in the UK on 19^th^ January 2022, designated as XE in Pango lineage, and monitored by UKHSA 38. XE acquired the gene encoding NSP1-6 from BA.1, a recombination breakpoint at nucleotide 11,537 on the NSP6, and a 3'-end including S and structural protein encoding genes from BA.2. In addition, three unique mutations that are not present in any of the BA.1 or BA.2 sequences are present in the XE genome. Two of these, NSP3 C3241T and NSP12 C14599T, are synonymous mutations, the latter being the only unlabelled private mutation, which may facilitate its identification. Another mutation NSP3 V1069I is a non-synonymous mutation responsible for the cleavage of viral polyproteins during replication 38,43. Regarding infectivity, XE showed a medium community growth rate (12.6%) above that of BA.2 according to the data uploaded by 30^th^ March 2022 on the UKHSA, and the infections were noted in patients of all age groups including paediatric patients, young adults, and individuals over 60 years of age 38,39. Furthermore, XE has been identified in at least 28 countries, including the United States of America, Israel, Japan, India, and Thailand after first case being detected in the UK; the spread across nations was linked with international travel 44. However, according to GISAID, XE prevalence has remained low and accounts for less than 1% of the total sequenced cases worldwide since its detection. The global average daily XE prevalence increased between January 19^th^ and late March 2022, marking the first peak that was followed by a transient decrease. The second peak, which was lower than the last one, was noted at the end of April 2022. Following this peak, the globally average daily XE prevalence declined steadily **(Figure 1 C)** 44,45.

Continuously emerging recombinant lineages have been detected in several settings. For example, the newly designated recombinant lineage XAZ was detected in at least 974 cases around the world 46. Further investigations are required to assess the transmission rate, disease severity, immunity, and vaccine effectiveness for these lineages. Notably, on 24^th^ October 2022, WHO discussed the public health implications of the rise in some of the Omicron variants, specifically the newly recombinant lineage XBB and its sublineages (indicated as XBB*), which are recombinants of BA.2.10.1 and BA.2.75 sublineages. As of epidemiological week 40 (3^rd^-9^th^ October 2022), based on the sequences submitted to GISAID, XBB* showed a global prevalence of 1.3% and has been detected in 35 countries. However, the available data do not suggest substantial differences in disease severity for XBB* infections, although the early evidence pointed at a higher reinfection risk compared to other circulating Omicron sublineages 47. Overall, we support two conclusions. First, genetic recombination is the key evolutionary mechanism that is continuously reshaping SARS-CoV-2 genetic diversity 48. Second, recombination is a widespread phenomenon in SARS-CoV-2 and therefore, although some of the previously detected SARS-CoV-2 recombinant lineages have shown limited circulation at a population level, it remains possible that highly-transmissible and/or pathogenic recombinant lineages emerge in the future 49. This underlines the need for continuous efforts to maintain an effective genomic surveillance of emerging recombinants to limit their spread.

## New waves of COVID-19 pandemic

Surveillance data indicates that since early July 2022, BA.4/5 and newly circulating descendent lineages/recombinant lineage are driving the new wave of the COVID-19 pandemic and have caused a global spike in infections at an unpresented speed, whereas the earlier dominant BA.2 and BA.2.12.1 are subsiding 50,51. These additional descendent lineages or recombinant lineage are derived from BA.4/5 including the BA.5.2, BA.5.2.6, BA.5.3.1.1.1.1.1 (BQ.1), BA.5.3.1.1.1.1.1.1 (BQ.1.1), BA.4.6, BA.5.2.1.7 (BF.7), and BA.5.1.7, and from BA.2 including BA.2.75 and BA.2.75.2, or from intra-BA.2 recombinant XBB; due to increased humoral immune escape, these variants exhibit successive or concurrent circulations 47,52.

Based on the epidemiological update on the week ending on 12^th^ November 2022, CDC Nowcast projections, estimate that the combined national proportion of lineages designated as Omicron will continue to be 100% with the predominant Omicron lineage BA.4/5 and its sublineages (including BQ.1.1, BQ.1, BF.7, BA.4.6, and BA.5.2.6), projected at 90.2% of circulating variants in the United States of America 53. In addition, according to country overview report from the European Centre for Disease Prevention and Control (ECDC) for weeks 42-43 (17^th^ -30^th^ October 2022), the estimated distribution in Europe was 79.5% for BA.5, 18.1% for BQ.1, 2.6% for BA.4, and 1.9% for BA.2.75, especially considering the increasing trend in the proportion of BQ.1 54. Furthermore, ECDC modelling forecasts predicted that BQ.1 and BQ1.1 would become the dominant SARS-CoV2 strains in EU/EEA from mid-November to the beginning of December 2022. This may contribute to an increase in the number of COVID-19 cases in the coming weeks to months, according to an epidemiological update by the ECDC 55. While there are no data on its severity or immune escape from studies conducted in humans, BQ.1 and its sublineages have shown a significant growth advantage over other circulating Omicron sublineages in many settings, including Europe and the US, and therefore warrant close monitoring 47. Furthermore, BQ.1 and XBB have been assigned UKHSA variant designations to facilitate continued studies 56. The circulating XBB is fuelling the surge in Singapore and is believed to be the causal factor behind the recent spike in cases 57. The first confirmed imported BA.5.2 case on mainland China was discovered on 4^th^ July 2022 from an international passenger coming from the U.S. to Beijing 58. Presently, BA.5.2 infection cases have escalated at an unpresented speed, affecting several areas on mainland China. A case report from Zhengzhou City Press Conference suggested that an asymptomatic individual infected with BA.5.2 spread the virus while passing other people in the toilet (only 80 seconds apart), indicating extremely high transmissibility. Moreover, two highly contagious variants, BF.7 and BA.5.1.7, have been detected in China that are highly infectious, show significantly high transmissibility, and have been described by the experts as dangerous for the public 59. Meanwhile, as COVID-19 cases are spiking, a warning has been issued against BF.7 and BA.5.1.7 by Chinese health agencies. Moreover, with present rise in its infection frequency, WHO has predicted that the BF.7 could be the next global dominant variant 60. Therefore, the epidemiologic surveillance as well as independent and comparative analyses for monitoring different Omicron variants are vital.

**Figure 3:**
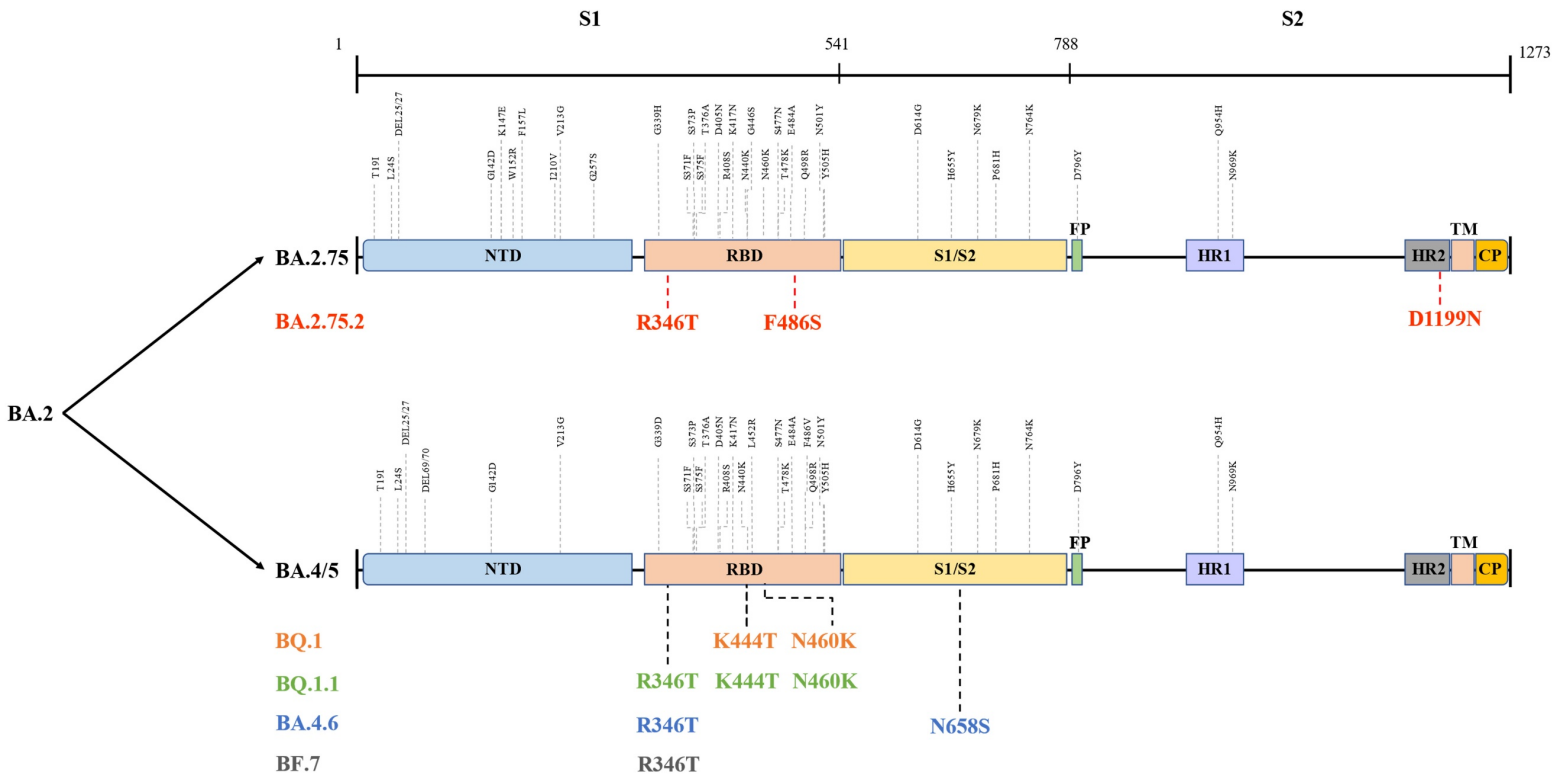
Different mutations in spike proteins of BA.2.75, BA.4/5, and their sublineages. Data derived from outbreak.info. On this figure, the colored mutations stand for additional mutations compared to the parental lineage. NTD: N-terminal domain; RBD: receptor-binding domain; FP: fusion peptide; HR1: heptad repeat 1; HR2: heptad repeat 2; TM: transmembrane region; IC: intracellular domain; CP: cytoplasmic Peptide.

The globally dominant Omicron variant is a cumulative outcome of S mutations, which caused the emergence of new sublineages and their descendent or recombinant lineages. Indeed, BA.5, BA.2.75, BA.2.75.2, BQ.1, BQ.1.1, BA.4.6, BF.7, and XBB harbour significant differences in their S region that confer them their unique properties **(Figure 3)** 61, which could influence their transmissibility by affecting unique host-immune evasion. For example, BA.5 carries L452R and F486V mutations that might tweak its ability to attach and latch onto host target cells and enhance host cell invasion as well as immune evasion 62. Research studies have indicated that BA.5 had a growth advantage over other Omicron sublineages and XD with a higher ability to escape immune response than the BA.1-3, BA.2.12.1, and XD 63. Similarly, molecular modelling studies pointed out that the N460K and F486S mutations could drive BQ.1 (N460K), BQ.1.1 (N460K) as well as BA.2.75.2 (F486S) toward evading neutralization by vaccine and infection-induced immunity 64. Notably, strong neutralization resistance to sera induced by BA.4/5 indicates a high chance of reinfection after recovery from BA.4/5 infection and reduced effectiveness of BA.4/5-bivalent-booster. Consistently, an experimental study from the US indicated that BA.5-bivalent-booster does not elicit strong immune responses capable of neutralizing BA.2.75.2 and BQ.1.1 65. Meanwhile, Miller et al. 66 revealed that after administration of the monovalent or bivalent mRNA vaccine boosters, neutralizing antibody (Nab) titres against BQ.1.1 were 7-fold lower than those against BA.5. Another live virus neutralization assay showed similar results 67. Furthermore, a recent preprint highlighted the significantly enhanced immune escape of XBB and BQ.1.1 using convalescent plasma following infection, vaccination, and existing antibody-based drugs, and that XBB driven by Y144del was the more antibody-evasive strain 68. Thus, although no data supported substantial differences in disease severity for XBB infections, its high reinfection risk conferred by increased immune evasion is concerning and needs further investigation. Besides, after measuring the neutralizing titres elicited by BA.4 and BA.5 harbouring Arg346 mutations, Jian et al. 69 concluded that BA.4.6 exhibited the greatest growth advantages among all variants as well as BA.4 and BA.5. These findings shed light on the immunologic context for the rapid expansion in prevalence of BA.4.6, BQ.1, BQ.1.1, and BA.2.75.2. In contrast, several studies did not report greater immune escape of BA.2.75 than that of BA.5, but showed higher hACE2-binding affinity and greater fusogenicity of BA.2.75, which may facilitate its spread in India 70-72. Similar to BA.2.75, BF.7 was more susceptible to neutralization than BQ.1.1 and BA.2.75.2, although it showed better immune evasion than its parental lineage BA.5 64,73. In brief, individuals harbouring humoral immunity induced by earlier Omicron lineages, other VOCs, or vaccination, arestill able to be infected with the newly emerging variants. Therefore, further studies and monitoring are required to determine whether the increased immune escape of these emerging lineages is sufficient to give rise to the next dominant variants.

## New vaccines and therapeutics

### New vaccines against Omicron

Currently, vaccination and therapeutic agents are the two most important tools against Omicron. Constantly S mutating of SARS-CoV-2 as well as prevalence of Omicron sublineages dictate to update a range of vaccine available for use worldwide to control the new variants. Currently, the following seven types of COVID-19 vaccines are being developed; protein subunit-, inactivated virus-, live attenuated virus-, virus like particle viral vector (non-replicating)-, viral vector (replicating)-, DNA-, and RNA-based vaccines 74. Several inactivated whole-virus and protein subunit vaccines need adjuvants to unleash the antibody-mediated immune response 75. As of 12^th^ November 2022, 3,441.126 million vaccine doses have been administered in mainland China; collectively, 90.65% adults aged over 60 years received at least one dose of vaccine, 86.38% were fully vaccinated, and 68.3% received a booster dose 76. On 4^th^ November 2022, German Chancellor Olaf Scholz, who made a one-day visit to Beijing, told reporters that China will make Pfizer-BioNTech COVID-19 vaccine available to foreigners living in the country 77.

On 13^th^ July 2022, the U.S. Food and Drug Administration issued an emergency use authorization (EUA) for the Novavax COVID-19 vaccine for the prevention of COVID-19 in individuals 18 years of age and older 78. Novavax COVID-19 vaccine contains Matrix-M adjuvant in addition to SARS-CoV-2 S protein to enhance the immune response in the vaccinated individuals. More recently, on 31st August 2022, the Food and Drug Administration recommended (FDA) authorized Moderna and Pfizer-BioNTech Bivalent COVID-19 vaccines for use as a single booster dose in individuals 18 and 12 years of age and older, respectively, at least two months following primary or booster vaccination. Other than the mRNA from the original SARS-CoV-2 strain, these bivalent vaccines also contain mRNA from Omicron lineages BA.4/5 and instruct the body cells to make the distinct S protein, which provides better protection against currently circulating variants 79. On 19^th^ October 2022, the U.S. FDA recommended individuals who were moderately or severely immunocompromised to receive one primary, one additional (monovalent mRNA COVID-19 vaccine), and one booster dose. Whereas others were recommended to receive one primary and one booster dose. The primary series dose and the additional boost must be separated by at least four weeks. A bivalent mRNA vaccine (“updated vaccine”) booster containing components of the original (ancestral) strain and the BA.4/BA.5 is recommended 80. This was supported by Adams et al., who observed that an mRNA vaccine booster dose provided additional benefit beyond a primary vaccine series alone for preventing hospital admissions with Omicron related COVID-19 during the first six months of 2022 in the US 81. In addition, a Phase Ⅱ/Ⅲ clinical trial (NCT04927065) reported that 50 μg bivalent Omicron-containing booster mRNA vaccine (named mRNA-1273.214, Moderna), which combines mRNA-1273.529 and mRNA-1273, elicited higher neutralizing antibody response against BA.4/5 than immune response elicited with mRNA-1273 28 days after immunization, without evident safety concerns. Bivalent booster mRNA vaccines induce enhanced and durable antibody responses and may be a new tool to control emerging Omicron sublineages and descendent lineages 82. On 15^th^ August 2022, Moderna, Inc. announced that the Medicines and Healthcare products Regulatory Agency (MHRA) in the UK has granted conditional authorization for the use of the mRNA-1273.214 (Spikevax Bivalent Original/Omicron) as a booster dose for active immunization to prevent COVID-19 in individuals 18 years of age and older 83. Furthermore, Moderna has completed regulatory submissions for mRNA-1273.214 in Australia, Canada, and the EU and expects further authorization decisions. In another recent study, Dai et al. reported a Phase Ш clinical trial (NCT04646590) of ZF2001 (a receptor-binding domain (RBD)-dimer-based vaccine), which showed that ZF2001 has more than 80% efficacy in preventing symptomatic SARS-CoV-2 infection 84. Moreover, Zhao et al. reported that neutralizing antibody titres against the BA.4 and BA.5 variants increased with increasing interval (4-6 months) between the second and third doses of ZF2001 85. In a preclinical assessment of a bivalent S-trimer vaccine based on SARS-CoV-2 Alpha and Beta variants (named SCTV01C) with a squalene-based oil-in-water adjuvant SCT-VA02B, Wang et al. demonstrated that the vaccine exhibited superior cross-neutralizing capacity against newly emerged BA.4/5 than WT (D614G)-based vaccine and had a favourable safety profile 86. As immunogenicity and durability of immunity against SARS-CoV-2 declines, more countries have advocated administration of the fourth vaccine dose. Importantly, a heterologous vaccine in form of sequential vaccination is recommended as a fourth booster dose to induce the immune system to produce effective “cross-immune memory” or “complementary-immune memory” to maximize protection against severe COVID-19 and death 87. Interestingly, to control the rapid emergence of next VOCs, scientific efforts would develop broad-spectrum vaccines against pan-SARS-CoV-2 or pan-sarbecovirus 88. Moreover, different types of nanovaccines have been developed to help overcome COVID-19 infections caused by Omicron variants and other VOCs in the future 89. However, the battle against SARS-CoV-2 is far from over due to its rapid evolution rate 90.

### Current therapeutics against COVID-19

So far, vaccine development has not been able to keep pace with SARS-CoV-2 evolution 84. Therefore, the focus of congoing research is to develop effective antivirals to induce an effective immune response against BA.4/BA.5 and their novel descendent lineages for overcoming COVID-19 epidemic. Regarding antiviral drugs, Evusheld developed by AstraZeneca is a combination of two long-acting antibodies tixagevimab and cilgavimab and has been authorized for prevention and protection against COVID-19 infection 91. On 29^th^ June 2022, FDA revised the Evusheld fact sheet for healthcare providers to recommend repeat administration every six months with a dose of 300 mg tixagevimab and 300 mg cilgavimab if continued protection was required. The previous fact sheet for healthcare providers did not give a specific recommendation on the dosing interval. FDA continues to monitor the neutralizing activity of Evusheld against emerging SARS-CoV-2 variants and will provide additional updates as needed 92. Takashita et al. 93 reported that the three small-molecule antiviral drugs remdesivir and molnupiravir (inhibitors of the RNA-dependent RNA polymerase of SARS-CoV-2), and nirmatrelvir (an inhibitor of the main protease) might have therapeutic value against BA.2.12.1, BA.4, and BA.5, and indicated that bebtelovimab was effective against BA.2.12.1, BA.4, and BA.5. On 25^th^ July 2022, azvudine, the first endogenous anti-SARS-CoV-2 oral drug, was conditionally approved by the National Medical Products Administration (NMPA) in China 94. Azvudine, a nucleoside analogue that inhibits viral RNA-dependent RNA polymerase (RdRp), can specifically act on the SARS-CoV-2 RdRp to inhibit viral replication, and shows strong targeting property. Azvudine is effective in clinically mild and severe patients, and the virus clearance time is approximately 5 days 95. In recent a prospective, open-label, randomized controlled trial (ChiCTR2200060292), Xu et al. 96 demonstrated that treatment with Reyanning (RYN) mixture accelerated virus clearance and promoted disease recovery in patients with asymptomatic and mild Omicron infections, and disease progression or serious adverse events were not observed. Pavan et al. 97 analysed the structural features of the S protein and Main Protease (Mpro) of XE, XD, and XF, and proposed the development of pan-coronaviral drugs that could prevent future coronavirus-associated pandemics. Given that some antiviral drugs can lose activity against circulating BA.5, COVID-19 convalescent plasma therapy will need to be revisited as a reliable therapeutic strategy in specific patient populations 98. On 3rd November 2022, the *Nature* journal published a multinational Delphi consensus to end the COVID-19 public health threat, and three of the highest-ranked recommendation calls were for the adoption of whole-society and whole-government approaches, while maintaining proven prevention measures using approaches that employ public health and financial support measures to complement vaccination 99.

## Conclusions

COVID-19 is an ongoing global public health emergency. New waves of Omicron infection demonstrate that COVID-19 is far from being over. As for now, it remains unclear when and where the next SARS-CoV-2 variant would appear 100. Furthermore, as SARS-CoV-2 continues to spread globally, it will inevitably overlap with different viral infections, which is a risk factor for adverse clinical outcomes. A recent study published in the *Journal of Infection* reported the first documented case of co-infection with monkeypox virus, SARS-CoV-2, and HIV virus. Thus, further studies are required to ascertain whether this combination may aggravate patient condition or worsen patient outcomes 101. A similar scenario was reported for a 40-year-old male patient who was co-infected with syphilis, HIV, and monkey-pox viruses and his nasal area underwent necrosis within three days, illustrating the potential severity of coinfection 102. Thus, although viral eradication is unlikely, it is important to devise comprehensive strategies that incorporate effective genomic surveillance of SARS-CoV-2 infections as well as advanced public health and preventive interventions for overcoming a global health challenge which appears to be in for a long haul.
